# Nano-Scale Characterization of a Piezoelectric Polymer (Polyvinylidene Difluoride, PVDF)

**DOI:** 10.3390/s8117359

**Published:** 2008-11-18

**Authors:** Hyungoo Lee, Rodrigo Cooper, Ke Wang, Hong Liang

**Affiliations:** 1 Department of Mechanical Engineering, Texas A&M University, College Station, Texas 77843-3123, USA; E-Mails: prettylhg@tamu.edu; rcooper@tamu.edu; 2 Department of Physics, Texas A&M University, College Station, Texas 77843-3123, USA; E-Mail: kephwk@tamu.edu;

**Keywords:** Polyvinylidene difluoride (PVDF), piezoelectricity, ferroelectricity, conductivity, atomic force microscope (AFM)

## Abstract

The polymer polyvinylidene difluoride (PVDF) has unique piezoelectric properties favorable for Micro-Electro-Mechanical Systems (MEMS) and Nano-Electro-Mechanical Systems (NEMS) applications. In the present research, we conducted nanometer-length scale characterization of this material using several high-resolution techniques. Specifically, we used an atomic force microscope (AFM) to study the nano-and microstructures of the PVDF under stress and to measure their nanoscale conductivity and piezoelectricity. We found that the surface morphology, electronic structure, and microstructure are profoundly affected under electrical potential. Such a behavior is important for the properties and performance of MEMS and NEMS.

## 1. Introduction

Piezoelectric materials play an important role for Micro-electro-mechanical systems (MEMS) and Nano-electro-mechanical systems (NEMS) [1, 2]. Polyvinylidene difluoride (PVDF) has been widely used in engineering applications due to its favorable chemical and mechanical properties [3-7]. Properties such as high piezoelectric coefficient, good flexibility, biocompatibility [8-10], low acoustic and mechanical impedance, and light weight, are especially unique for MEMS applications. The polymer PVDF is one of the most widely used piezoelectric materials in the fluoropolymer family. Its piezoelectricity was discovered in 1969 [11]. However, the mechanism of piezoelectricity has not been clearly explained [12-14]. This uncertainty has hindered the development of MEMS or NEMS.

Many researchers have attempted to explain the origin of the piezoelectricity of the polymer [15-19] and to investigate the structure change of PVDF due to electric field [19] and temperature [20]. Recently, development in new characterization techniques, particularly nanoscale analysis, has made it possible to bring new insights from the piezoelectricity measurements. One of these techniques is Atomic Force Microscopy, which enables characterization of surface morphology at the nano-scale. Using this technique, we have investigated the relationship between the piezoelectric properties and structures of PVDF. An electrical field was applied to the PVDF samples. The effects of the external electrical voltage on the sample surface were observed using an atomic force microscope (AFM). The responding time of the PVDF samples to mechanical stress was studied.

## 2. Experiments

### 2.1. Materials

This study was performed using three different samples of uniaxially oriented polyvinylidene difluoride (PVDF) films. The original PVDF film samples were uniaxially stretched to obtain the polar β-phase. The samples were examined for polarity (β-phase, TTT type) and piezoelectric coefficient (23pC/N). Using a metal evaporator, the metal coatings were deposited on the films as electrodes. Two samples with thickness of 110 μm and one with 52 μm were coated with 28 μm of Ag, 600 Å of NiCu, and 150 nm of Au, respectively. The size of all samples was 3 cm in length and 1cm width.

### 2.2. AFM and the System Setup

**Figure 1.**
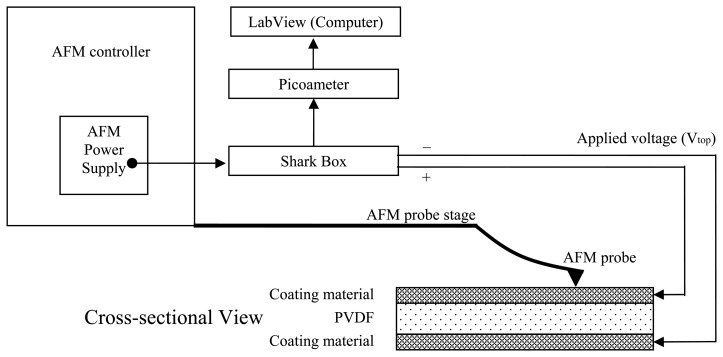
Diagram for the experimental setup and characterization. This work was done with a Si3N4 probes. The external electrical potentials were supplied to the PVDF samples throughout the shark box which is a splitter for electrical potentials and current.

A home-built splitter, i.e., a Shark box, was attached to an AFM. The splitter distributes electrical potentials and passes a current from samples to a picoameter. The splitter was powered through the AFM's built-in power supply as shown in Figure 1. The sample surface is scanned with a standard Si_3_N_4_ probe. The surface profile is measured against the electrical potential with respect to the principal direction (d_31_) of the PVDF films.

When bent, a PVDF sample generates a micro-ampere current flowing to a picoameter. A computer is connected to the picoameter to record the output current using the LabView software. In this case, conductive AFM probes were used.

### 2.3. Motorized Linear Stage (MLS)

**Figure 2.**
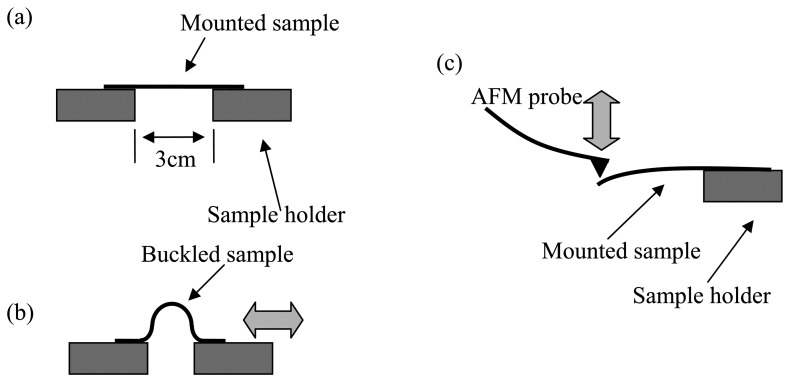
Simple diagrams of Motorized Linear Stage (MLS) (a and b) and AFM setup (c) to apply mechanical stresses on a sample.

A motorized linear stage (MLS) was used to characterize the responding time of the dipoles in PVDF. The coated PVDF samples were placed on the MLS from one end to the other as shown in Figure 2(a) and (b). With one of the sample holders fixed in a stationary position, the other holder moved reciprocally at a frequency of 4Hz in a stroke length of 3cm.

The reciprocal motion induced buckling over PVDF samples. Figure 2(c) shows the AFM setup applying a force on one end. The PVDF sample produced an electrical potential due to the piezoelectric property. The voltage generated was recorded using the LabView through a picoameter. MSL and AFM probe bending tests were conducted with respect to the principal direction (d_31_) of the PVDF films.

## 3. Results

**Figure 3.**
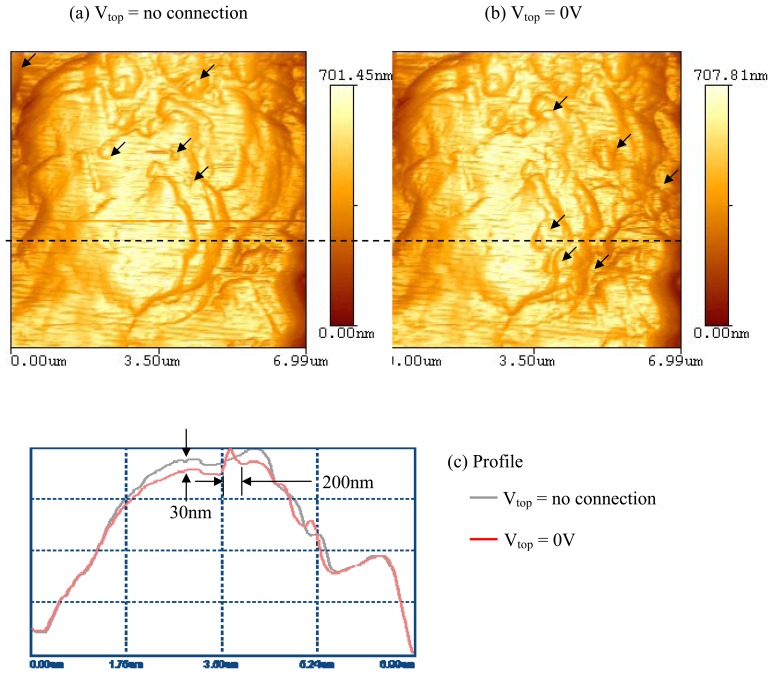
AFM images for the PVDF sample surface which was 110μm thick with 28μm Ag coated without (a) or with (b) connection of electric field. (c) The profiles of the two scans along the dashed line

Scans of a PVDF sample, with the thickness of 110μm and an Ag-coating of 28μm, were conducted using the AFM with a standard Si_3_N_4_ probe. Figure 3(a) shows a scanned image of the sample prior to connecting the Shark box.

The same region of the sample was scanned with the leads of the shark box connected to the PVDF as shown in Figure 1 while an applied electrical potential was at 0V. The profiles of the two scans along the dashed line are shown in Figure 3(c). It is seen that at the zero potential, the surface profile underwent a morphology change resulting in an increase in roughness.

**Figure 4.**
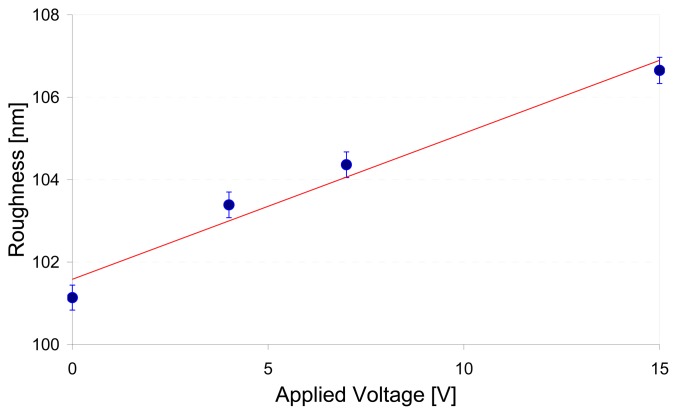
Roughness change with the applied voltage.

As the electric potential on the PVDF is increased in discrete steps of 0, 4, 7, and 15 V, the roughness (R_a_) is seen to increase in a nearly linear manner as shown in Figure 4. A similar process was conducted for a PVDF sample of 110μm thickness and 600Å NiCu coat to characterize its surface morphology with external electrical potentials of 0 and 5V.

**Figure 5.**
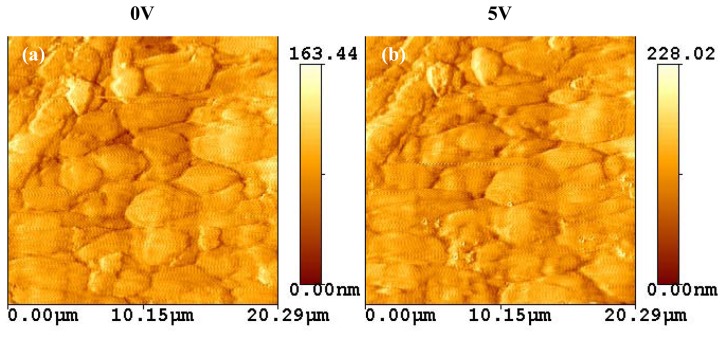
The texture change of a PVDF surface. The PVDF sample of 110μm thickness with 600Å NiCu coated was characterized for its surface with external electrical potentials, 0V (a) and 5V (b).

As shown in Figure 5, the surface texture was squeezed with the application of 5V. This is believed to be due to the dipole realignment resulting in an increased surface height.

**Figure 6.**
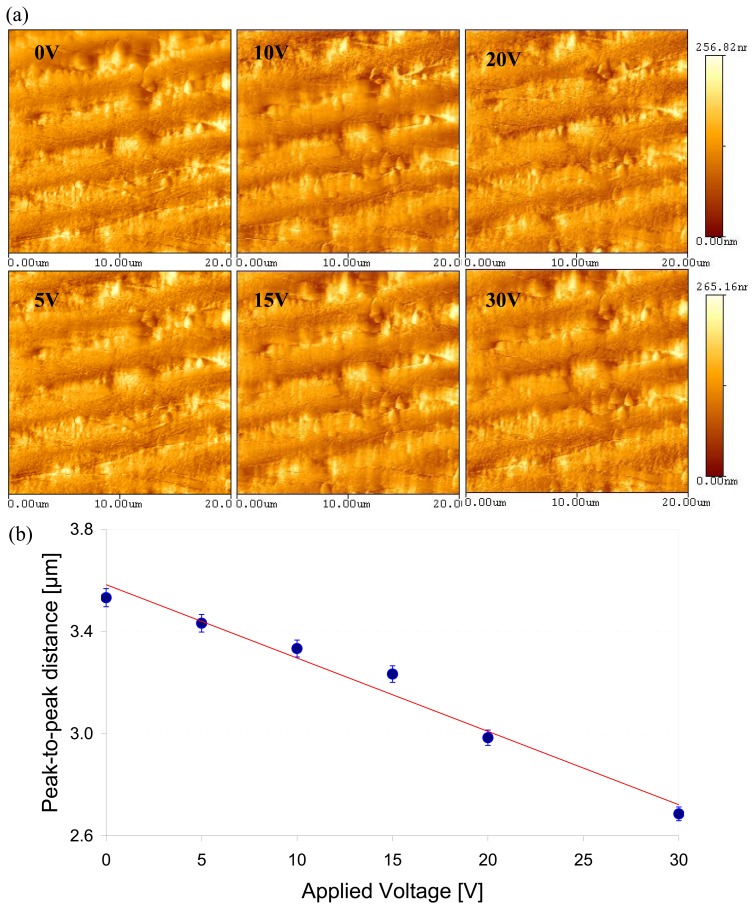
Investigation of the surface change rate with the applied voltage. (a) AFM images for a PVDF sample which was 52μm thickness with 150nm Au coated. (b) The change of the peak-to-peak distance (D_pp_) with the external electrical potentials.

A PVDF sample with the 52 μm thickness and a 150 nm-Au-coating, was used to determine the amount of deformation caused by the applied voltage. This sample showed an original wavy surface when no voltage was applied. As a voltage was increased, the amount of squeezing deformation was observed and calculated through measuring the peak-to-peak distance (D_pp_) of the wave surface. Figure 6(a) shows AFM images of the sample under different voltages. Figure 6(b) displays the change of the D_pp_ due to external electrical potentials. As the applied electrical potential increased, the D_pp_ was decreased with the rate of 28.7nm/V. The slope of the gray line in the figure is equal to the effective piezoelectric coefficient. It is noted that without an electrical potential, the dipoles inside of the films were not aligned. They were randomly oriented and their polarity were cancelled each other so that the films were at 0V. When the 0V was applied externally, the dipoles were aligned; negative pole was oriented to a positive charge, and positive pole to negative charge. With “zero” volt, the net voltage due to alignment was too small to be seen. However, the alignment was observed in the morphology change.

## 4. Discussion

Some polymers have showed transformation on its surface due to external stimuli [21-23]. The surface of an electro-sensitive polymer changed polymer chains and alignment with an applied electrical field [24]. Figures 3, 5, and 6 demonstrate the effect of inverse piezoelectricity due to the alignment of the dipoles in response to an applied electric potential. As a result, the potential caused the change of a surface morphology. Furthermore, the alignment of the dipoles was a temporary response to a voltage and returned to their original state after the voltage was removed. This effect is caused by lamellae (usually 20nm thick) of crystalline embedded within amorphous regions (chain-folded model) of the samples. It was reported that one a 3D polymer was fixed at its ends, the local intra- and inter-chain associated orientational-deformational interactions could induce spontaneous ordering [25]. The lamellae were randomly oriented with no electric potentials present in the sample. As an electric potential was applied, the lamellae realigned to orient their dipole angle and moment correspondingly. The higher the applied potential, the greater the realignment, and in such, the greater the peak-to-valley distance. The rate of the D_pp_ change was about 1nm per 2V.

**Figure 7.**
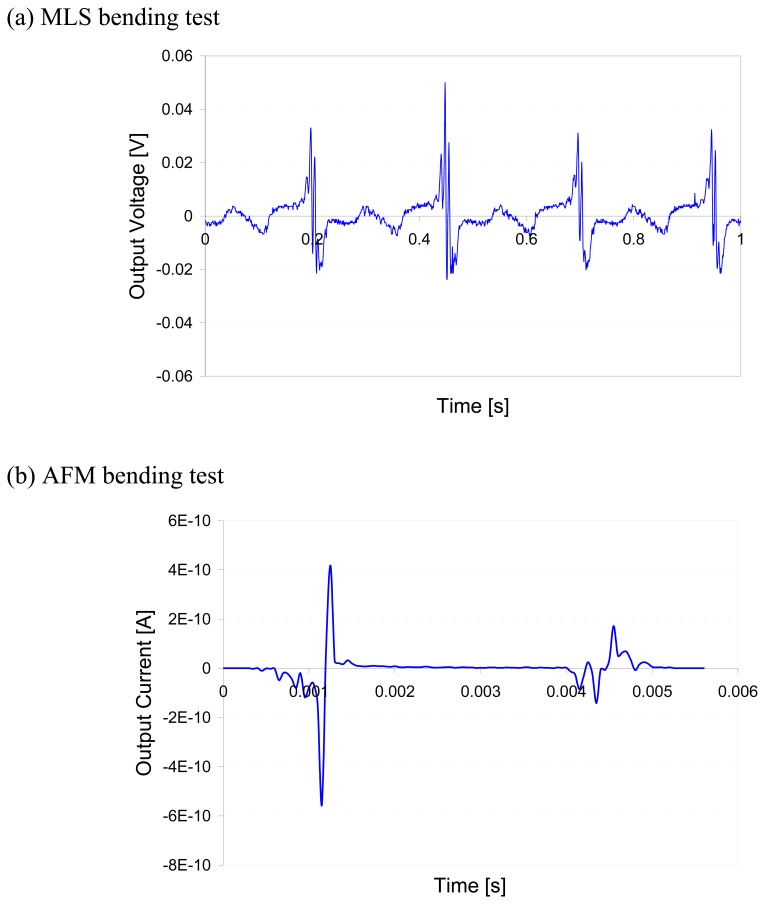
The results of (a) Motorized Linear Stage (MLS) and (b) AFM tests showing the output have similar behavior at different scales. The MLS test was to fix two ends of the sensor while the AFM was on a sensor that was fixed at one end only with manual bending.

In order to understand the piezoelectric behavior at different length scales, we compared the charge output under stresses at nanometer and millimeter scales. The first measurement was conducted by applying external mechanical stress on the samples with the MLS shown in Figure 2. As the distance between the sample holders decreases, a mechanical stress is applied to the sample resulting in the alignment of the lamellae which produces a voltage due to the alignment of the dipoles. Figure 7(a) shows the response of such a piezo sensor. The voltage produced by the PVDF is proportional to the amount of stress applied to the sample which is proportional to the distance traveled by the MLS holders. Since the sample holder is moving in a reciprocating motion, there should be no movement at its maximum and minimum deflection thus not causing further alignment. The AFM was used to test the output of a piezo sensor when a down force was applied at one end of the polymer. Although the units of the MLS and AFM were different, the trend of their outputs was the same. This means that the localized dipole alignment is correlated with the global behavior. When an external force was applied, local or global, the PVDF produces a charge and subsequently a morphological change. The effects of electrical potential on microstructures and phase transformation of PVDF have been discussed earlier [26]. Since our focus of this research is on the surface morphological study of a piezo sensor, we will not discuss this aspect in detail.

## 5. Conclusions

Metalized PVDF samples were characterized using an AFM and an external MLS in order to study the mechanisms of piezoelectric effects. Results showed that under an external potential, the surface roughness was increased. Under stress, an output was generated that is scale independent. Such variations are important for design consideration of MEMS devices and their sensitivity.
